# Variant Location Is a Novel Risk Factor for Individuals With Arrhythmogenic Cardiomyopathy Due to a Desmoplakin (*DSP*) Truncating Variant

**DOI:** 10.1161/CIRCGEN.121.003672

**Published:** 2022-12-29

**Authors:** Edgar T. Hoorntje, Charlotte Burns, Luisa Marsili, Ben Corden, Victoria N. Parikh, Gerard J. te Meerman, Belinda Gray, Ahmet Adiyaman, Richard D. Bagnall, Daniela Q.C.M. Barge-Schaapveld, Maarten P. van den Berg, Marianne Bootsma, Laurens P. Bosman, Gemma Correnti, Johan Duflou, Ruben N. Eppinga, Diane Fatkin, Michael Fietz, Eric Haan, Jan D.H. Jongbloed, Arnaud D. Hauer, Lien Lam, Freyja H.M. van Lint, Amrit Lota, Carlo Marcelis, Hugh J. McCarthy, Anneke M. van Mil, Rogier A. Oldenburg, Nicholas Pachter, R. Nils Planken, Chloe Reuter, Christopher Semsarian, Jasper J. van der Smagt, Tina Thompson, Jitendra Vohra, Paul G.A. Volders, Jaap I. van Waning, Nicola Whiffin, Arthur van den Wijngaard, Ahmad S. Amin, Arthur A.M. Wilde, Gijs van Woerden, Laura Yeates, Dominica Zentner, Euan A. Ashley, Matthew T. Wheeler, James S. Ware, J. Peter van Tintelen, Jodie Ingles

**Affiliations:** Department of Genetics, University Medical Centre Groningen, University of Groningen (E.T.H., G.J.t.M., J.D.H.J.).; Netherlands Heart Institute, Utrecht, the Netherlands (E.T.H., L.P.B., L.L., J.P.v.T.).; Agnes Ginges Centre for Molecular Cardiology at Centenary Institute (C.B., B.G., R.D.B., C.S.).; Faculty of Medicine and Health (C.B., B.G., R.D.B., J.D., C.S., L.Y., J.I.).; Department of Cardiology, Royal Prince Alfred Hospital, Sydney, Australia (C.B., B.G., C.S., L.Y., J.I.).; Department of Clinical Genetics, Amsterdam University Medical Centre, location AMC, University of Amsterdam, the Netherlands (L.M., J.P.v.T.).; Clinique de Génétique, CHU Lille, Lille, France (L.M.).; National Heart and Lung Institute and MRC London Institute of Medical Science, Imperial College London and Cardiovascular Research Centre, Royal Brompton and Harefield NHS Foundation Trust, London, UK (B.C., A.L., N.W., J.S.W.).; Stanford Centre for Inherited Cardiovascular Disease, Department of Medicine, Stanford University School of Medicine, CA (V.N.P., C.R., E.A.A., M.T.W.).; Department of Cardiology, Isala Heart Center, Zwolle (A.A.).; Department of Clinical Genetics, Leiden University Medical Centre (D.Q.C.M.B.-S., A.M.v.M.).; Department of Cardiology, University of Groningen, University Medical Centre Groningen (M.P.v.d.B., G.v.W.).; Department of Cardiology, University of Leiden, Leiden University Medical Centre (M.B.).; Department of Cardiology, University of Utrecht (L.P.B.); Adult Genetics Unit, Royal Adelaide Hospital and Faculty of Health and Medical Sciences, University of Adelaide (G.C.).; Victor Chang Cardiac Research Institute, Sydney (D.F.).; Department of Diagnostic Genomics, PathWest Laboratory, Medicine WA, Redlands, Australia (M.F.).; Department of Cardiology, Haga Teaching Hospital, the Hague (A.D.H.).; Department of Genetics, University of Utrecht, University Medical Centre Utrecht, the Netherlands (F.H.M.v.L., J.P.v.T.).; Department of Clinical Genetics, Radboud University Medical Centre, Nijmegen, the Netherlands (C.M.).; Department of Clinical Genetics, Children’s Hospital Westmead, Sydney, Australia (H.J.M.).; Department of Clinical Genetics, Erasmus University Medical Centre, Rotterdam, the Netherlands (R.A.O.).; Genetic Services of Western Australia, Perth, Australia (N.P.).; Department of Radiology and Nuclear Medicine, Amsterdam University Medical Centre, Amsterdam, the Netherlands (R.N.P.).; Department of Cardiology and Department of Genomic Medicine, Royal Melbourne Hospital (T.T., J.V., D.Z.).; Faculty of Medicine, Dentistry and Health Sciences, The University of Melbourne, Australia (J.V., D.Z.).; Department of Cardiology, Cardiovascular Research Institute Maastricht (CARIM) (P.G.A.V.).; Department of Clinical Genetics, Laboratory Clinical Genetics, Maastricht University Medical Centre (A.v.d.W.).; Department of Clinical and Experimental Cardiology, Heart Centre, Amsterdam University Medical Centre, location AMC, the Netherlands (A.S.A., A.A.M.W.).; Cardio Genomics Program at Centenary Institute, The University of Sydney (L.Y., J.I.).; Centre for Population Genomics, Garvan Institute of Medical Research and UNSW Sydney (J.I.).; Centre for Population Genomics, Murdoch Children’s Research Institute, Melbourne, Australia (J.I.).

**Keywords:** cardiomyopathies, primary, death, sudden cardiac, desmoplakins, genetic testing

## Abstract

Truncating variants in desmoplakin (*DSP*tv) are an important cause of arrhythmogenic cardiomyopathy; however the genetic architecture and genotype-specific risk factors are incompletely understood. We evaluated phenotype, risk factors for ventricular arrhythmias, and underlying genetics of *DSP*tv cardiomyopathy.

**Methods:**

Individuals with *DSP*tv and any cardiac phenotype, and their gene-positive family members were included from multiple international centers. Clinical data and family history information were collected. Event-free survival from ventricular arrhythmia was assessed. Variant location was compared between cases and controls, and literature review of reported *DSP*tv performed.

**Results:**

There were 98 probands and 72 family members (mean age at diagnosis 43±8 years, 59% women) with a *DSP*tv, of which 146 were considered clinically affected. Ventricular arrhythmia (sudden cardiac arrest, sustained ventricular tachycardia, appropriate implantable cardioverter defibrillator therapy) occurred in 56 (33%) individuals. *DSP*tv location and proband status were independent risk factors for ventricular arrhythmia. Further, gene region was important with variants in cases (cohort n=98; Clinvar n=167) more likely to occur in the regions resulting in nonsense mediated decay of both major *DSP* isoforms, compared with n=124 genome aggregation database control variants (148 [83.6%] versus 29 [16.4%]; *P*<0.0001).

**Conclusions:**

In the largest series of individuals with *DSP*tv, we demonstrate that variant location is a novel risk factor for ventricular arrhythmia, can inform variant interpretation, and provide critical insights to allow for precision-based clinical management.

Desmoplakin is a plakin family protein that anchors the desmosome to intermediate filaments and is abundant in tissues with greater mechanical stress such as the epidermis and myocardium.^1,2^ Genetic variants in the gene encoding desmoplakin (*DSP*) cause a range of cardio-cutaneous phenotypes, including arrhythmogenic cardiomyopathy (ACM), striate palmoplantar keratoderma, and lethal acantholytic epidermolysis bullosa in more severe cases.^3^ Truncating variants (*DSP*tv) that lead to putative loss of function via haploinsufficiency of the protein have been previously reported as causative of disease.^2^ *DSP*-null mice show extensive disruption of the cytoarchitecture and cell resilience in skin and heart tissue, with death in early development.^4^

Arrhythmogenic right ventricular cardiomyopathy (ARVC), the right dominant sub-form of ACM,^2,5^ is characterized by progressive loss and fibrofatty replacement of the ventricular myocardium.^6^ Diagnosis of ARVC can be challenging and 2010 Task Force Criteria consider electrical, structural (imaging and histological), and genetic characteristics.^7^ Historically, clinical descriptions of *DSP*tv were often based on ARVC cohorts, though growing recognition of left ventricular (LV) involvement has necessitated a shift to a broader phenotype description, ACM,^8^ encompassing left dominant arrhythmogenic cardiomyopathy and biventricular disease, with new Padua criteria proposed.^9^ Dilated cardiomyopathy and left dominant arrhythmogenic cardiomyopathy lie on a spectrum, with overlap in molecular causes. More recently, *DSP* has been definitely associated with both ARVC and Dilated cardiomyopathy by international gene curation expert panels^10,11^ In one of the largest studies to date, clinical characteristics of *DSP* variants in a population of 44 probands and 63 family members were reported as a distinct ACM characterized by LV fibrosis, myocardial inflammation and high incidence of ventricular arrhythmias.^12^ Biallelic *DSP* variants can give rise to Carvajal syndrome, characterized by woolly hair, palmoplantar keratoderma and development of ACM in childhood, and often due to homozygous or compound heterozygous *DSP*tv affecting the C-terminus.^13^

The N-terminal globular head of *DSP* is important in desmosome organization by binding plaque proteins such as plakophilin and plakoglobin, while the central rod domain contains a coiled-coil region.^14^ The C-terminal contains 3 plakin repeat domains, required for alignment and binding of intermediate filaments.^15^ Two predominant isoforms exist due to alternate splicing: *DSPI* (which is the longest isoform) and *DSPII* (which has a shortened central rod domain). DSPI and DSPII are expressed in equivalent levels in epidermis; however, DSPI is more prevalent in myocardium.^16^ Differences between the 2 isoforms relate to the rod domain size, considered important for self-association and formation of homo-dimers.^17^

Here we report an international cohort of individuals with a *DSP*tv. We describe the phenotype spectrum of *DSP*tv cardiomyopathy, family history characteristics, and provide insights into the genetic architecture of *DSP*tv cardiomyopathy and its relation to clinical phenotype.

## Methods

Data are available by request to the corresponding author and adhering to site ethical approval. All aspects of the study were performed according to institutional human research ethics committee approval according to the local sites. Institutional ethics approval was granted by individual sites; including Sydney Local Health District, Royal Prince Alfred Hospital, Australia; Royal Brompton & Harefield Hospitals Cardiovascular Biobank (National Research Ethics Service), UK. Waiver of consent was granted at Stanford School of Medicine Internal Review Board, USA. All individual-level data were de-identified.

Detailed methods are available in the Supplemental Material.

## Results

### Study Population

Overall there were 98 probands (mean age at diagnosis 42±18 years; 59% women) and 72 family members identified (mean age at diagnosis 45±19 years; 61% women; Table 1). There were 95 probands with a cardiomyopathy and 3 with a primary cutaneous phenotype. Among family members, 48 of 72 were deemed affected, including 5 with a predominantly cutaneous phenotype. In total, 146 individuals were considered affected, including cardiomyopathy, ventricular arrhythmia and cutaneous phenotypes.

**Table 1.**
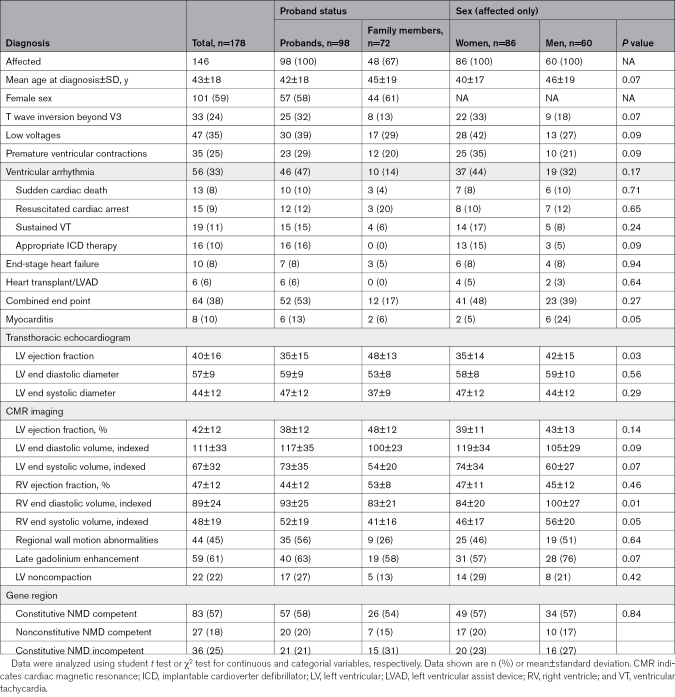
Clinical Characteristics by Proband Status and Sex

### Sex Differences

Women were over-represented compared with men among affected individuals (86 [59%] versus 60 [41%]; Table 1). There was no difference in mean age at diagnosis between women and men (40±17 years versus 46±19 years; *P*=0.07). Myocarditis was more frequent in men (2/43 [5%] versus 6/25 [24%]; *P*=0.046) but this was not always reliably reported. Women had reduced LV ejection fraction on transthoracic echocardiography, but not cardiac magnetic resonance imaging (CMR) derived LV ejection fraction. Men had greater indexed right ventricular end diastolic volume (84±20 versus 100±27; *P*=0.01). No differences in clinical outcomes were reported between sexes. There was a comparable distribution of variants by gene region for men and women, as well as probands and affected relatives.

### Electrophysiological Characteristics

There was a high rate of ventricular arrhythmia occurring in 56 (33%) individuals, including 46 (47%) probands and 10 (14%) family members. Ventricular arrhythmia included sudden cardiac death (SCD) in 13 (8%; 10 probands), resuscitated cardiac arrest in 15 (9%; 12 probands), appropriate implantable cardioverter defibrillator therapy in 16 (10%; 16 probands), and sustained ventricular tachycardia in 19 patients (11%; 15 probands); including 10 (14%) family members, and with some experiencing multiple events. Six probands experienced 2 ventricular arrhythmia episodes, initially having sustained ventricular tachycardia (n=4) or resuscitated cardiac arrest (n=2), followed by appropriate implantable cardioverter defibrillator therapy. SCD or resuscitated cardiac arrest was the presenting symptom in 24 (14%; 20 probands) patients. T wave inversion beyond V3 occurred in 33 (24%), low voltages in 47 (35%) and premature ventricular contractions in 56 (33%).

### Imaging Characteristics

Echocardiographic and CMR characteristics are shown in Table 1. Signs of LV noncompaction were reported (n=22), with 6 having a ratio of noncompacted to compacted layer >2.3 on CMR. Four probands were reported to have hypertrophic cardiomyopathy (HCM) with ages at diagnosis ranging from 58 to 83 years, and LV hypertrophy measuring 26 mm, 16 mm, and an apical pattern in 2. *DSP*tv are not established as associated with HCM, and we consider it unlikely that these variants are causal for HCM for these 4 cases, but are reported as they met the pre-specified eligibility criteria. Late gadolinium enhancement (LGE) was reported in 59 (61%), and end-stage heart failure was reported in 10 (8%) patients. Two women developed disease while pregnant, 1 showed impaired LV function (LV ejection fraction <45%) at 32 weeks of gestation, while the other developed narrow complex tachycardia at 38 weeks of gestation with subsequent echocardiogram showing a dilated and impaired LV. A further 2 women developed disease during the postpartum period. Finally, another patient who died suddenly during pregnancy was identified to be positive for Parvovirus B19 on postmortem Parvo-polymerase chain reaction in myocardial tissue. Myocarditis was reported in 7 individuals on CMR (and another on postmortem investigation).

### Genetic Analysis

A total of 69 distinct *DSP*tv were identified in the 98 probands (Table S1). Among the 69 *DSP*tv, there were 31 small insertions or deletions leading to a frameshift and downstream premature termination codon, 25 nonsense variants, 12 canonical splice-site altering variants and a large deletion of exons 5–24. Eleven (16%) variants were classified as pathogenic, 57 (83%) were classified as likely pathogenic and 1 (1%) was classified as a variant of uncertain significance. Two probands had a diagnosis of cardiomyopathy, with woolly hair and keratoderma (OMIM 605676) and were compound heterozygous, each carrying a *DSP*tv (p.Arg2229Serfs*32 or p.Tyr28Alafs*66) and a *DSP* splice site variant (the same c.273+5G>A in both). This splice site variant has an allele count of 79 in genome aggregation database (gnomAD), with an allele frequency of 0.028% and considered a variant of uncertain significance under a recessive inheritance model.

### *DSP*tv Location

**Figure 1.**
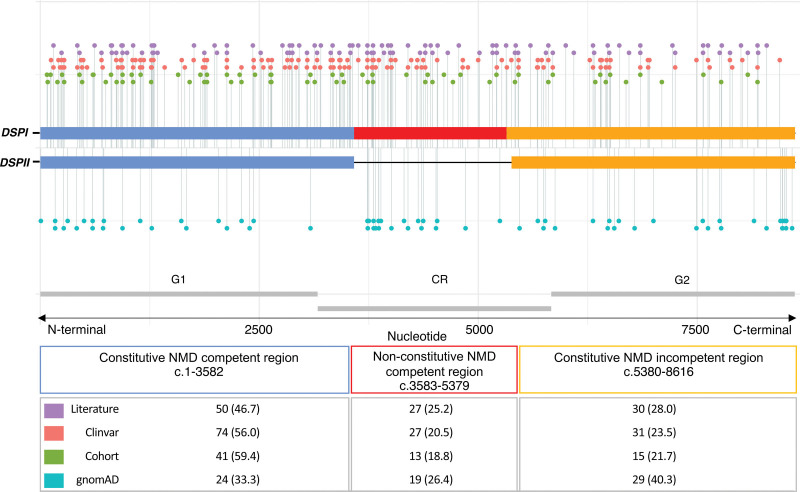
**Linear topology schematic showing distribution of *DSP*tv across key gene regions of major isoforms *DSPI* and *DSPII*.** Case variants (Cohort, Clinvar, and Literature) are shown above and control variants below the line. The number of unique variants (per proband) from each source are shown in the table. CR indicates central fibrous rod domain; DSPtv, desmoplakin truncating variant; G1, globular 1; G2, globular 2; gnomAD, gnome aggregation database; and NMD, nonsense mediated decay.

We investigated whether case and control variants localized to the specified gene regions, constitutive nonsense mediated decay (NMD) competent, nonconstitutive NMD-competent and constitutive NMD-incompetent (Figure 1). Pathogenic and likely pathogenic *DSP*tv submitted to ClinVar, as well as variants described above in the international cohort were included, giving a total of 265 cases. This included 69 unique *DSP*tv identified in 98 individuals in the international cohort and 134 unique *DSP*tv from 167 cases reported in ClinVar (Table S2). One variant reported in ClinVar was excluded from analysis given it resided in the small overlap region of exon 23 which is both nonconstitutive and predicted NMD-incompetent due to being <55 bp upstream of the last exon junction (*DSP*: c.5327_5330del; p.Glu1776Glyfs). Another was excluded after it was identified as a ClinVar entry for one of the cohort cases. Literature cases are shown in Figure 1 but not included in the analysis due to unquantified sample overlap. Variants observed in cases were compared with 72 unique *DSP*tv observed as 124 alleles in gnomAD controls (Table S3). Case variants were more frequently seen in the constitutive NMD-competent region compared with controls. Across the 3 gene regions (constitutive NMD-competent, nonconstitutive NMD-competent and constitutive NMD-incompetent, respectively), *DSP*tv were seen in 148 (56%), 59 (22%) and 58 (22%) cases compared with *DSP*tv observed in controls 29 (23%), 28 (23%) and 67 (54%), overall *P*<0.0001.

Clinical characteristics of patients with *DSP*tv in the 3 gene regions are shown in Table 2. Overall there were few significant differences between the patient groups based on gene region. Age at diagnosis was significantly younger in those with *DSP*tv in both NMD-competent regions (constitutive and nonconstitutive). Further, there was a greater risk of ventricular arrhythmia and risk of the combined end point in those with *DSP*tv in the constitutive and nonconstitutive NMD-competent regions.

**Table 2.**
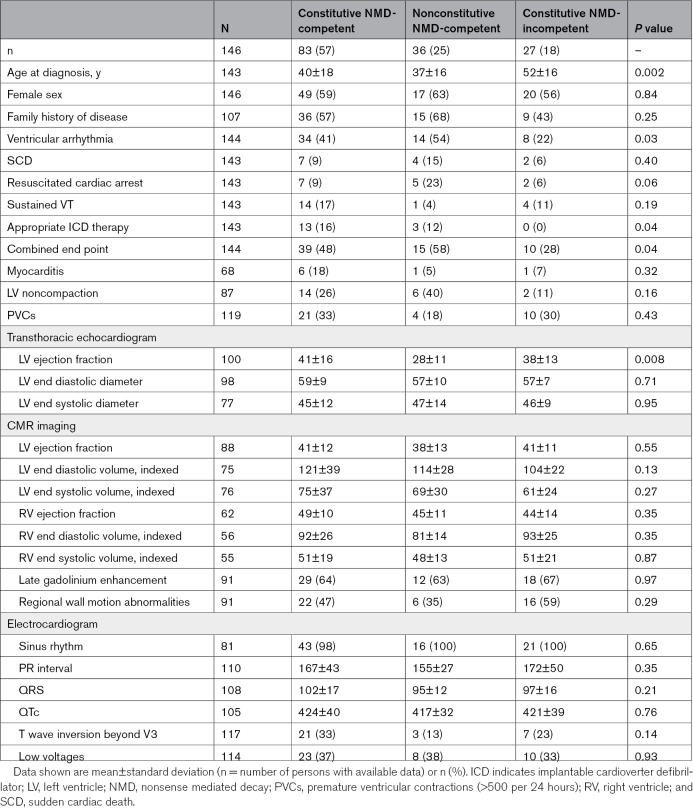
Cardiac Investigation of Affected Individuals With *DSP*tv by Gene Region

### Event-Free Survival From Ventricular Arrhythmia Based on Gene Region

**Figure 2.**
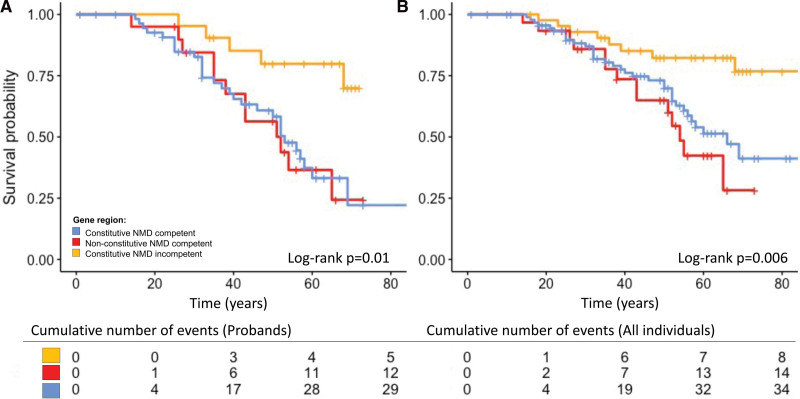
**Independent life-time risk factor for ventricular arrhythmia.** (**A**) Gene region including probands only. (**B**) Gene region including probands and affected family members. Time is given in years. NMD indicates nonsense mediated decay.

Information with regard to occurrence of ventricular arrhythmia or censoring was available for 167 individuals. There were 56 probands and family members who experienced a ventricular arrhythmia during their lifetime. Univariable Cox proportional hazards models showed gene region and proband status as significantly associated with worse survival from ventricular arrhythmias (Table 3; Figure 2). Adjusting for other variables, variants in the constitutive NMD competent region (HR, 2.8 [95% CI, 1.3–6.0]; *P*=0.01), nonconstitutive NMD-competent region (HR, 3.2 [95% CI 1.3–7.9]; *P*=0.009) and proband status (HR, 3.3 [95% CI, 1.7–6.6]; *P*=0.0006) remained significant independent life-time risk factors for ventricular arrhythmia (Table 3).

**Table 3.**
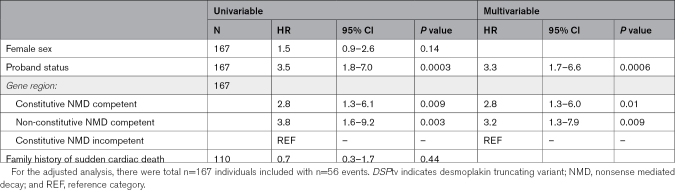
Lifetime Risk Factors for Ventricular Arrhythmia for Individuals With a *DSP*tv

### Cutaneous Phenotype

Cutaneous abnormalities were not systematically reported; however, notably 1 family with a *DSP*tv in the nonconstitutive NMD-competent region (cardiac isoform, *DSPI*) had an affected relative with hyperkeratosis and cardiomyopathy. An additional 13 individuals with *DSP*tv in the constitutive NMD-competent region and 5 in the constitutive NMD-incompetent region were reported with overt cardio-cutaneous features noted at clinical review. In 8 patients (5%; 3 probands) only cutaneous abnormalities were reported, and were the sole finding in 1 family following an autosomal dominant inheritance pattern.

### Postmortem Findings and Cardiac Transplant Histology

Thirteen patients (8%; 10 probands) presented with SCD (Table S4). In all 13, a postmortem investigation was performed. The mean age at death was 26±11 years. Where recorded, the activity at time of death varied from exercise through to sleep. No decedent had a pre-morbid diagnosis of a cardiac condition. Nine decedents received a postmortem diagnosis of ARVC or probable ARVC. There was LV involvement in all cases and fibrosis and fatty infiltration commonly reported.

Two patients underwent a heart transplant due to end stage heart failure. Biventricular involvement was observed in both hearts, as were signs of LV noncompaction. One heart showed ARVC with septal involvement and replacement fibrosis in both ventricles and septum. The other heart showed LV noncompaction with notable right ventricular involvement consisting of fatty changes and atrophy.

### Family History Characteristics

Among the probands, 49 (51%) had a documented family history of cardiomyopathy, while 16 (17%) had a family history of a suspicious SCD under the age of 40 years. Of the 72 family members with positive gene results included, 48 (67%) had overt disease, while 24 (33%) remained asymptomatic (mean age of 49±22 years and 15 [63%] were women). There were 8 family members aged 60 years or older (60–86 years; 5 women) with no clinical evidence of disease, suggesting incomplete penetrance. By gene region, there was no statistical difference in the proportion of probands with a positive family history (constitutive NMD-competent 27 [49%], nonconstitutive NMD-competent 13 [65%], constitutive NMD-incompetent 9 [43%], *P*=0.33).

### Literature Review of Previously Reported *DSP*tv

Three hundred and fifteen studies were identified, 240 were screened, and 185 full texts were assessed for eligibility (85 were excluded from the final qualitative synthesis, including 66 that did not report any *DSP* variant, 2 where phenotype was not provided, 2 with no full-text article available, and 1 review; Figure S1). Of the 98 studies (describing both disease and genotype-first cohorts) included in the final selection, a total of 105 *DSP*tv in 143 probands from apparently unrelated families were reported, including 57 nonsense, 42 frameshift, and 6 splice site variants (Tables S5–S7). All reported variants were absent or very rare (allele count ≤2) in gnomAD and were classified as pathogenic or likely pathogenic. One variant (p.Thr2104fs*12) was present 13 times in gnomAD, however has strong evidence of pathogenicity and reported in a compound heterozygous state.

Both dominant and recessive patterns of inheritance of *DSP*tv were reported. Cascade genetic testing to confirm autosomal dominant inheritance was reported for only 19 *DSP*tv (dominant *DSP*tv) in 22 families. Families reported with autosomal dominant inheritance commonly demonstrated adult age of onset, incomplete penetrance and variable clinical expression. Of the 105 reported *DSP*tv, 26 were only identified in affected individuals with homozygous or compound heterozygous inheritance. Four *DSP*tv co-occurred in trans with one of 3 missense *DSP* variants (p.Ala2655Asp, p.Arg2366Cys, and p.Asn287Lys), each of which involved highly conserved residues within globular heads, are absent in gnomAD, and classified as likely pathogenic. There were 16 individuals with 23 *DSP*tv identified to have autosomal recessive disease, either homozygous (n=9) or compound heterozygous (n=7). In just those variants identified in a homozygous state there was only 1 (11%) in the constitutive NMD-competent region, 5 (56%) in the nonconstitutive NMD-competent region and 3 (33%) in the constitutive NMD-incompetent region.

## Discussion

**Figure 3.**
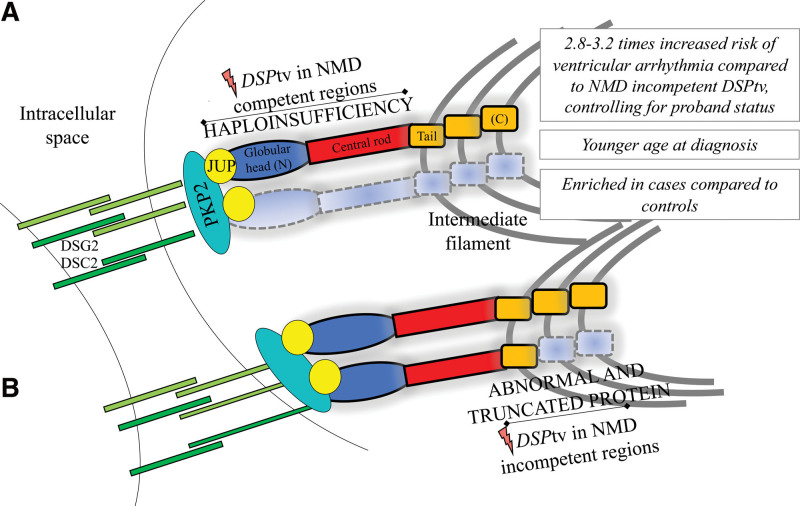
**Summary of the key findings and illustration of the impact of DSPtv location on protein expression (for DSPI).** DSC indicates desmocollin-2; DSG2, desmoglein-2; JUP, plakoglobin; PKP2, plakophilin-2; and NMD, nonsense mediated decay.

*DSP*tv lead to a distinct cardiomyopathy characterized by LV involvement and a high-risk of ventricular arrhythmia and SCD. We present a large international series of cases with *DSP*tv and demonstrate that the location of the *DSP*tv is a novel risk factor for ventricular arrhythmia (Figure 3). Truncating variants in the constitutive NMD-competent region were enriched in cases compared with controls, and predicted to result in NMD and haploinsufficiency of both DSPI and DSPII. Our findings highlight the importance of personalized medicine and the move towards gene-guided management of patients in the future.

Ventricular arrhythmias occur frequently in patients with *DSP*tv cardiomyopathy, with one previous study reporting 23% presenting with SCD events.^18^ In our cohort, 47% of probands had ventricular arrhythmia either at presentation or during follow-up. In addition, 14% of relatives experienced ventricular arrhythmia, including 7% as their initial presenting symptom. This included 2 probands who presented with resuscitated cardiac arrest without any overt structural abnormalities of the heart, supporting the notion that life-threatening electrical phenotype can precede overt cardiac structural disease.^19–21^ While we were unable to robustly ascertain clinical risk factors due to the large proportion of cases who presented with ventricular arrhythmia (ie, without necessary pre-event clinical data), a recent series of 107 patients with any *DSP* variants (n=30 events) showed ventricular arrhythmias were associated with reduced LV ejection fraction, while premature ventricular contractions (>500 beats in 24 hours), LGE, and right ventricular dysfunction were not shown to be associated with ventricular arrhythmia.^12^ Family history of SCD has not previously been evaluated in this group, and we showed it is not associated with ventricular arrhythmia in our population.

Prior observation that *DSP*tv are predominantly associated with a left dominant form of ACM^2,5,22^ is in line with our findings. Recent examples of *DSP*tv presenting as recurrent myocarditis and acute myocardial infarction-like events have also been reported.^23,24^ Women were overrepresented in our population, but otherwise shared similar clinical characteristics compared with men, except reduced indexed right ventricular end diastolic diameter on CMR. This finding is in contrast to other reported inherited cardiomyopathy patient cohorts, where a higher prevalence of men is often reported.^25–27^ Of note, a recent report of ARVC presenting as clinical myocarditis showed disproportionately more women, with 10/11 having *DSP*tv.^28^ *DSP*tv cardiomyopathy patients frequently had low QRS voltage and negative T waves beyond V3. Low QRS voltage in limb leads have previously been shown to be associated with the presence and amount of LGE in a study of patients with ARVC.^8^ Regional wall motion abnormalities on CMR and epicardial to mid wall LGE patterns in the LV were frequently seen in our cohort. Septal LGE frequently occurs in patients with left dominant arrhythmogenic cardiomyopathy,^15^ and recent work has shown patients with *DSP* and *FLNC* ACM are more likely to have LGE, often with a ring-like pattern, compared with other Dilated cardiomyopathy genotypes.^29^ Four probands were reported to have HCM, however it should be noted that all 4 probands were men, presenting in older age and 3 had mild LV hypertrophy, all characteristics previously described in the nonfamilial sub-group of HCM.^30^ Previous assessment of the clinical validity of *DSP* variants causing HCM failed to identify sufficient evidence of gene-disease association.^31^ While our finding remains unclear, it seems reasonable to consider these clinical diagnoses as unrelated to the *DSP*tv.

A recent systematic evaluation of cutaneous abnormalities among *DSP*tv showed all patients expressed some degree of skin or hair abnormalities, except those with *DSP*tv in the nonconstitutive NMD-competent region (cardiac isoform, DSPI).^32^ Interestingly, we report 1 proband and their affected relative with palmoplantar keratoderma, with a *DSP*tv in the nonconstitutive NMD-competent region. Another study reported 10% of *DSP*tv had cutaneous disease only, while 12% were reported to have LV dominant ACM and cutaneous disease.^5^

We show *DSP*tv localized to the constitutive NMD-competent region, corresponding to the N-terminal globular head, were enriched in patients compared with controls, and this finding was replicated in the variants identified through literature review. This region plays a critical role in organization and assembly of the desmosomal complex by binding with plakophilin and plakoglobin. One previous report of *DSP* missense variants in patients with a clinical diagnosis of ARVC suggested a potential “hotspot” N-terminal region, with 8/17 (47%) missense variants localized to the N-terminal compared with 1/28 (4%) of controls (*P*<0.0008).^33^ Further, they concluded *DSP*tv were significantly more prevalent in ARVC cases than controls. Indeed, a recent study also showed clustering of missense variants in the N-terminal, but reported *DSP*tv to be more evenly distributed across the gene,^12^ potentially limited by sample size. Another showed enrichment of missense variants in the spectrin repeat domain, which is part of the constitutive NMD competent region.^34^ While it seems likely that truncating variants in the NMD-incompetent region escape NMD and have a later onset and less deleterious impact, functional work to date has shown highly variable pattern of protein expression representing both haploinsufficiency and dominant negative effects.^35^ Our literature review identified biallelic *DSP*tv localized more often to the constitutive NMD-incompetent region compared with dominant *DSP*tv, suggesting that single heterozygous *DSP*tv are more likely to cause disease when occurring in the NMD-competent regions. Further, very few cases with homozygous variants in the constitutive NMD-competent region have been reported, with 1 example of a sib pair with severe lethal acantholytic epidermolysis bullosa, who died at 1 and 3 days respectively.^36^ It seems unlikely these infants were DSP null, given *DSP* knockout mice show embryonic lethality,^4^ suggesting expression of low-level truncated protein may be able to rescue the phenotype to some degree. Taken together, identification of a *DSP*tv in the NMD-competent regions should be considered important and may prompt gene and disease-specific adaptation and use of the ACMG/AMP (American College of Medical Genetics and Genomics and Association for Molecular Pathology) criteria.^37^ We suggest *DSP*tv in this region be allocated very strong level of evidence, PVS1, when considering pathogenicity, when seen in an individual with a well characterized and concordant phenotype.

## Study Limitations

This was a large retrospective cohort study, and while it was an international effort, differences in practices and data collection by site meant some variables were incomplete. Furthermore, the event rate and data missingness precluded more detailed risk factor analyses. Diagnosis was made by the referring clinician and most recruitment was from specialized tertiary referral centers and therefore likely represents more severe phenotypes. The literature review was limited by publication bias, and inconsistent reporting of clinical, family and genetic information.

## Conclusions

We present a large international series of individuals with *DSP*tv and show gene region is a novel risk factor, specifically *DSP*tv leading to predicted NMD of truncated protein and haploinsufficiency of DSPI and/or DSPII is a risk factor for ventricular arrhythmias. By sub-typing disease by genotype there is increasing ability to offer precision medicine-based advice and therapies, and thereby improved outcomes for patients and their families.

## Article Information

### Sources of Funding

Dr Burns is the recipient of an Australia Postgraduate Award (APA). Dr Bagnall is supported by a grant from New South Wales Health. Dr Semsarian is the recipient of a National Health and Medical Research Council (NHMRC) Practitioner Fellowship (#1154992). Dr Ware is supported by the Wellcome Trust, the Medical Research Council (UK), the British Heart Foundation, the NIHR Royal Brompton Biomedical Research Unit, and the NIHR Imperial College Biomedical Research Centre. Drs van Tintelen, Wilde, Volders, van den Berg, and Hoorntje acknowledge the support from the Netherlands Cardiovascular Research Initiative, an initiative with support of the Dutch Heart Foundation (2014-40 DOSIS; 2012-10 PREDICT; 2018-30 PREDICT2; 2015-12 eDETECT). Dr Ingles is the recipient of an NHMRC Career Development Fellowship (#1162929). The other authors report no conflicts.

### Disclosures

Dr Ingles receives research grant support from Bristol Myers Squibb, unrelated to this study. Dr Ware reports research grant support and consultancy fees from Bristol Myers Squibb, unrelated to this study. Dr Reuter is a consultant for My Gene Counsel. Dr Wheeler is a stockholder of Personalis Inc. The remaining authors have nothing to disclose.

### Supplemental Material

Supplemental Material

Tables S1–S7

Figure S1

References^37–42^

## Supplementary Material


