# Variant Location Is a Novel Risk Factor for Individuals with Arrhythmogenic Cardiomyopathy Due to a Desmoplakin (*DSP*) Truncating Variant

**DOI:** 10.1161/CIRCGEN.121.003672

**Published:** 2022-12-29

**Authors:** Edgar T. Hoorntje, Charlotte Burns, Luisa Marsili, Ben Corden, Victoria N. Parikh, Gerard J. te Meerman, Belinda Gray, Ahmet Adiyaman, Richard D. Bagnall, Daniela Q.C.M. Barge-Schaapveld, Maarten P. van den Berg, Marianne Bootsma, Laurens P. Bosman, Gemma Correnti, Johan Duflou, Ruben N. Eppinga, Diane Fatkin, Michael Fietz, Eric Haan, Jan D.H. Jongbloed, Arnaud D. Hauer, Lien Lam, Freyja H.M. van Lint, Amrit Lota, Carlo Marcelis, Hugh J. McCarthy, Anneke M. van Mil, Rogier A. Oldenburg, Nicholas Pachter, R. Nils Planken, Chloe Reuter, Christopher Semsarian, Jasper J. van der Smagt, Tina Thompson, Jitendra Vohra, Paul G.A. Volders, Jaap I. van Waning, Nicola Whiffin, Arthur van den Wijngaard, Ahmad S. Amin, Arthur A.M. Wilde, Gijs van Woerden, Laura Yeates, Dominica Zentner, Euan A. Ashley, Matthew T. Wheeler, James S. Ware, J. Peter van Tintelen, Jodie Ingles

**Affiliations:** 1 Dept of Genetics, Univ Medical Ctr Groningen, Univ of Groningen; 2 Netherlands Heart Inst, Utrecht, the Netherlands; 3 Agnes Ginges Ctr for Molecular Cardiology at Centenary Inst,; 4 Faculty of Medicine & Health,; 5 Dept of Cardiology, Royal Prince Alfred Hospital, Sydney, Australia; 6 Dept of Clinical Genetics, Amsterdam Univ Medical Ctr, location AMC, Univ of Amsterdam, the Netherlands; 7 Clinique de Génétique, CHU Lille, Lille, France; 8 National Heart and Lung Inst & MRC London Inst of Medical Science, Imperial College London; 9 Cardiovascular Rsrch Ctr, Royal Brompton & Harefield NHS Foundation Trust, London, UK; 10 Stanford Centre for Inherited Cardiovascular Disease, Dept of Medicine, Stanford Univ School of Medicine, CA; 11 Dept of Cardiology, Isala Heart Center, Zwolle; 12 Dept of Clinical Genetics, Leiden Univ Medical Ctr, Leiden; 13 Dept of Cardiology, Univ of Groningen, Univ Medical Ctr Groningen; 14 Dept of Cardiology, Univ of Leiden, Leiden Univ Medical Ctr; 15 Dept of Cardiology, Univ of Utrecht,; 16 Adult Genetics Unit, Royal Adelaide Hospital & Faculty of Health & Medical Sciences, Univ of Adelaide; 17 Victor Chang Cardiac Rsrch Inst, Sydney; 18 Dept of Diagnostic Genomics, PathWest Laboratory, Medicine WA, Redlands, Australia; 19 Dept of Cardiology, Haga Teaching Hospital, the Hague; 20 Dept of Genetics, Univ of Utrecht, Univ Medical Ctr Utrecht, the Netherlands; 21 Dept of Clinical Genetics, Radboud Univ Medical Ctr, Nijmegen, the Netherlands; 22 Dept of Clinical Genetics, Children’s Hospital Westmead, Sydney, Australia; 23 Dept of Clinical Genetics, Erasmus Univ Medical Ctr, Rotterdam, the Netherlands; 24 Genetic Services of Western Australia, Perth, Australia; 25 Dept of Radiology & Nuclear Medicine, Amsterdam Univ Medical Ctr, Amsterdam, the Netherlands; 26 Dept of Cardiology & Dept of Genomic Medicine, Royal Melbourne Hospital; 27 Faculty of Medicine, Dentistry & Health Sciences, The Univ of Melbourne, Australia; 28 Dept of Cardiology, Cardiovascular Rsrch Inst Maastricht (CARIM); 29 Dept of Clinical Genetics, Laboratory Clinical Genetics, Maastricht Univ Medical Ctr; 30 Dept of Clinical & Experimental Cardiology, Heart Ctr, Amsterdam Univ Medical Ctr, location AMC, Amsterdam, the Netherlands; 31 Cardio Genomics Program at Centenary Inst, The Univ of Sydney; 32 Ctr for Population Genomics, Garvan Inst of Medical Rsrch & UNSW Sydney; 33 Ctr for Population Genomics, Murdoch Children’s Rsrch Inst, Melbourne, Australia

**Keywords:** Arrhythmogenic cardiomyopathy, desmoplakin, genetic testing, sudden cardiac death

## Abstract

**Background:**

Truncating variants in desmoplakin (*DSP*tv) are an important cause of arrhythmogenic cardiomyopathy (ACM), however the genetic architecture and genotype-specific risk factors are incompletely understood. We evaluated phenotype, risk factors for ventricular arrhythmias, and underlying genetics of *DSP*tv cardiomyopathy.

**Methods:**

Individuals with *DSP*tv and any cardiac phenotype, and their gene-positive family members were included from multiple international centers. Clinical data and family history information were collected. Event-free survival from ventricular arrhythmia was assessed. Variant location was compared between cases and controls, and literature review of reported *DSP*tv performed.

**Results:**

There were 98 probands and 72 family members (mean age at diagnosis 43 ± 18 years, 59% female) with a *DSP*tv, of which 146 were considered clinically affected. Ventricular arrhythmia (sudden cardiac arrest, sustained ventricular tachycardia, appropriate implantable cardioverter defibrillator therapy) occurred in 56 (33%) individuals. *DSP*tv location and proband status were independent risk factors for ventricular arrhythmia. Further, gene region was important with variants in cases (cohort n=98, Clinvar n=167) more likely to occur in the regions resulting in nonsense mediated decay of both major *DSP* isoforms, compared to n=124 gnomAD control variants (148 [83.6%] versus 29 [16.4%], p<0.0001).

**Conclusions:**

In the largest series of individuals with *DSP*tv, we demonstrate variant location is a novel risk factor for ventricular arrhythmia, can inform variant interpretation, and provide critical insights to allow precision-based clinical management.

## Nonstandard Abbreviations and Acronyms

DSP: desmoplakin
DSPtv: desmoplakin truncating variant
ACM: arrhythmogenic cardiomyopathy
DCM: dilated cardiomyopathy
ARVC: arrhythmogenic right ventricular cardiomyopathy
HCM: hypertrophic cardiomyopathy
LDAC: left dominant arrhythmogenic cardiomyopathy
LV: left ventricular
LVEF: left ventricular ejection fraction
CMR: cardiac magnetic resonance imaging
LVNC: left ventricular noncompaction
NMD: nonsense mediated decay
ICD: implantable cardioverter defibrillator
RV: right ventricle
LGE: late gadolinium enhancement
SCD: sudden cardiac death
ACMG/AMP: American College of Medical Genetics and Genomics and Association for Molecular Pathology

## Introduction

Desmoplakin is a plakin family protein that anchors the desmosome to intermediate filaments and is abundant in tissues with greater mechanical stress such as the epidermis and myocardium.^1, 2^ Genetic variants in the gene encoding desmoplakin (*DSP*) cause a range of cardio-cutaneous phenotypes including arrhythmogenic cardiomyopathy (ACM), striate palmoplantar keratoderma and lethal acantholytic epidermolysis bullosa in more severe cases.^3^ Truncating variants (*DSP*tv) that lead to putative loss of function (LOF) via haploinsufficiency of the protein have been previously reported as causative of disease.^2^ *DSP*-null mice show extensive disruption of the cytoarchitecture and cell resilience in skin and heart tissue, with death in early development.^4^

Arrhythmogenic right ventricular cardiomyopathy (ARVC), the right dominant sub-form of ACM,^2, 5^ is characterised by progressive loss and fibrofatty replacement of the ventricular myocardium.^6^ Diagnosis of ARVC can be challenging and 2010 Task Force Criteria consider electrical, structural (imaging and histological) and genetic characteristics.^7^ Historically, clinical descriptions of *DSP*tv were often based on ARVC cohorts, though growing recognition of left ventricular (LV) involvement has necessitated a shift to a broader phenotype description, ACM,^8^ encompassing left dominant arrhythmogenic cardiomyopathy (LDAC) and biventricular disease, with new Padua criteria proposed.^9^ Dilated cardiomyopathy (DCM) and LDAC lie on a spectrum, with overlap in molecular causes. More recently, *DSP* has been definitely associated with both ARVC and DCM by international gene curation expert panels^10, 11^ In one of the largest studies to date, clinical characteristics of *DSP* variants in a population of 44 probands and 63 family members were reported as a distinct ACM characterised by LV fibrosis, myocardial inflammation and high incidence of ventricular arrhythmias.^12^ Biallelic *DSP* variants can give rise to Carvajal syndrome, characterised by woolly hair, palmoplantar keratoderma and development of ACM in childhood, and often due to homozygous or compound heterozygous *DSP*tv affecting the C-terminus.^13^

The N-terminal globular head of *DSP* is important in desmosome organisation by binding plaque proteins such as plakophilin and plakoglobin, while the central rod domain contains a coiled-coil region.^14^ The C-terminal contains three plakin repeat domains, required for alignment and binding of intermediate filaments.^15^ Two predominant isoforms exist due to alternate splicing, *DSPI* which is the longest isoform and *DSPII* which has a shortened central rod domain. DSPI and DSPII are expressed in equivalent levels in epidermis, however DSPI is more prevalent in myocardium.^16^ Differences between the two isoforms relate to the rod domain size, considered important for self-association and formation of homo-dimers.^17^

Here we report an international cohort of individuals with a *DSP*tv. We describe the phenotype spectrum of *DSP*tv cardiomyopathy, family history characteristics, and provide insights into the genetic architecture of *DSP*tv cardiomyopathy and its relation to clinical phenotype.

## Methods

Data are available by request to the corresponding author and adhering to site ethical approval. All aspects of the study were performed according to institutional human research ethics committee approval according to the local sites. Institutional ethics approval was granted by individual sites; including Sydney Local Health District, Royal Prince Alfred Hospital, Australia; Royal Brompton & Harefield Hospitals Cardiovascular Biobank (National Research Ethics Service), UK. Waiver of consent was granted at Stanford School of Medicine Internal Review Board, USA. All individual-level data were de-identified.

## Results

### Study population

Overall there were 98 probands (mean age at diagnosis 42 ± 18 years, 59% female) and 72 family members identified (mean age at diagnosis 45 ± 19 years, 61% female; Table 1). There were 95 probands with a cardiomyopathy and 3 with a primary cutaneous phenotype. Among family members, 48/72 were deemed affected, including 5 with a predominantly cutaneous phenotype. In total, 146 individuals were considered affected including cardiomyopathy, ventricular arrhythmia and cutaneous phenotypes.

### Sex differences

Females were over-represented compared to males among affected individuals (86 [59%] versus 60 [41%]; Table 1). There was no difference in mean age at diagnosis between females and males (40 ± 17 years versus 46 ± 19 years, p=0.07). Myocarditis was more frequent in males (2/43 [5%] versus 6/25 [24%], p=0.046) but this was not always reliably reported. Women had reduced LV ejection fraction (LVEF) on transthoracic echocardiography, but not cardiac magnetic resonance imaging (CMR) derived LVEF. Men had greater indexed right ventricular (RV) end diastolic volume (84 ± 20 versus 100 ± 27, p=0.01). No difference in clinical outcomes were reported between sexes. There was a comparable distribution of variants by gene region for men and women, as well as probands and affected relatives.

### Electrophysiological characteristics

There was a high rate of ventricular arrhythmia occurring in 56 (33%) individuals, including 46 (47%) probands and 10 (14%) family members. Ventricular arrhythmia included sudden cardiac death (SCD) in 13 (8%; 10 probands), resuscitated cardiac arrest in 15 (9%; 12 probands), appropriate implantable cardioverter defibrillator (ICD) therapy in 16 (10%; 16 probands) and sustained ventricular tachycardia in 19 patients (11%; 15 probands); including 10 (14%) family members, and with some experiencing multiple events. Six probands experienced two ventricular arrhythmia episodes, initially having sustained ventricular tachycardia (n=4) or resuscitated cardiac arrest (n=2), followed by appropriate ICD therapy. SCD or resuscitated cardiac arrest was the presenting symptom in 24 (14%; 20 probands) patients. T wave inversion beyond V3 occurred in 33 (24%), low voltages in 47 (35%) and premature ventricular contractions in 56 (33%).

### Imaging characteristics

Echocardiographic and CMR characteristics are shown in Table 1. Signs of LV noncompaction (LVNC) were reported (n=22), with 6 having a ratio of noncompacted to compacted layer >2.3 on CMR. Four probands were reported to have hypertrophic cardiomyopathy (HCM) with ages at diagnosis ranging from 58-83 years, and LV hypertrophy measuring 26mm, 16mm, and an apical pattern in two. *DSP*tv are not established as associated with HCM, and we consider it unlikely that these variants are causal for HCM for these 4 cases, but are reported as they met the pre-specified eligibility criteria. Late gadolinium enhancement (LGE) was reported in 59 (61%), and end-stage heart failure was reported in 10 (8%) patients. Two females developed disease while pregnant, one showed impaired LV function (LVEF <45%) at 32 weeks of gestation, while the other developed narrow complex tachycardia at 38 weeks of gestation with subsequent echocardiogram showing a dilated and impaired LV. A further two women developed disease during the postpartum period. Finally, another patient who died suddenly during pregnancy was identified to be positive for Parvovirus B19 on postmortem Parvo-polymerase chain reaction in myocardial tissue. Myocarditis was reported in 7 individuals on CMR (and another on postmortem investigation).

### Genetic analysis

A total of 69 distinct *DSP*tv were identified in the 98 probands (Supplementary Table I). Among the 69 *DSP*tv, there were 31 small insertions or deletions leading to a frameshift and downstream premature termination codon, 25 nonsense variants, 12 canonical splice-site altering variants and a large deletion of exons 5-24. Eleven (16%) variants were classified as pathogenic, 57 (83%) were classified as likely pathogenic and 1 (1%) was classified as a variant of uncertain significance. Two probands had a diagnosis of cardiomyopathy, with woolly hair and keratoderma (OMIM 605676) and were compound heterozygous, each carrying a *DSP*tv (p.Arg2229Serfs*32 or p.Tyr28Alafs*66) and a *DSP* splice site variant (the same c.273+5G>A in both). This splice site variant has an allele count of 79 in gnomAD, with an allele frequency of 0.028% and considered a variant of uncertain significance under a recessive inheritance model.

### *DSP*tv location

**Figure 1:**
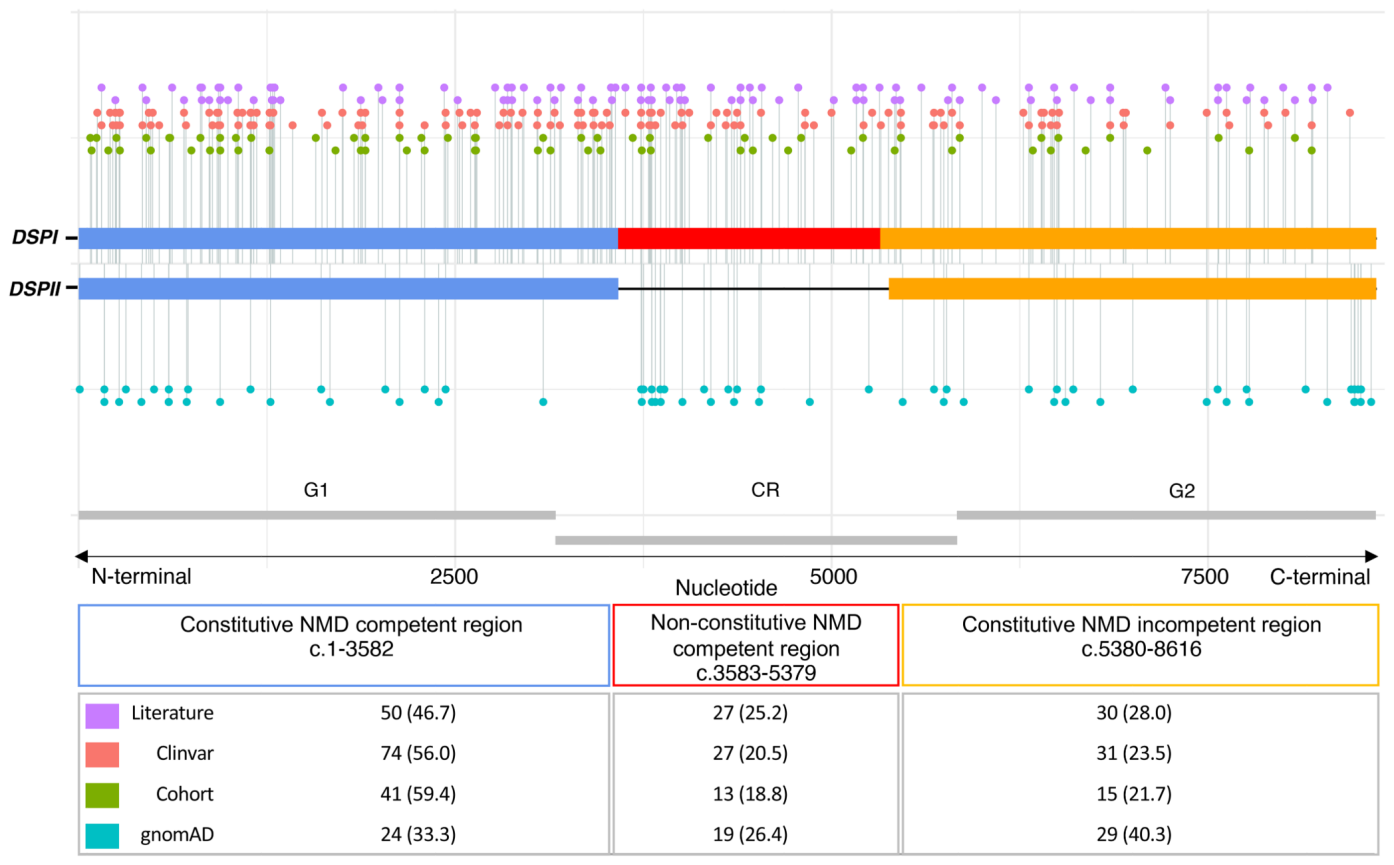
Linear topology schematic showing distribution of *DSP*tv across the key gene regions of *DSPI* and *DSPII*. Case variants (Cohort, Clinvar and Literature) are shown above and control variants below the line. The number of unique variants (per proband) from each source are shown in the table. Abbreviation: NMD, nonsense mediated decay.

We investigated whether case and control variants localised to the specified gene regions, constitutive NMD-competent, non-constitutive NMD-competent and constitutive NMD-incompetent (Figure 1). Pathogenic and likely pathogenic *DSP*tv submitted to ClinVar, as well as variants described above in the international cohort were included, giving a total of 265 cases. This included 69 unique *DSP*tv identified in 98 individuals in the international cohort and 134 unique *DSP*tv from 167 cases reported in ClinVar (Supplementary Table II). One variant reported in ClinVar was excluded from analysis given it resided in the small overlap region of exon 23 which is both non-constitutive and predicted NMD-incompetent due to being <55 bp upstream of the last exon junction *(DSP:* c.5327_5330del, p.Glu1776Glyfs). Another was excluded after it was identified as a ClinVar entry for one of the cohort cases. Literature cases are shown in Figure 1 but not included in the analysis due to unquantified sample overlap. Variants observed in cases were compared to 72 unique *DSP*tv observed as 124 alleles in gnomAD controls (Supplementary Table III). Case variants were more frequently seen in the constitutive NMD-competent region compared to controls. Across the 3 gene regions (constitutive NMD-competent, non-constitutive NMD-competent and constitutive NMD-incompetent, respectively), *DSP*tv were seen in 148 (56%), 59 (22%) and 58 (22%) cases compared to *DSP*tv observed in controls 29 (23%), 28 (23%) and 67 (54%), overall p<0.0001.

Clinical characteristics of patients with *DSP*tv in the three gene regions are shown in Table 2. Overall there were few significant differences between the patient groups based on gene region. Age at diagnosis was significantly younger in those with *DSP*tv in both NMD-competent regions (constitutive and non-constitutive). Further, there was a greater risk of ventricular arrhythmia and risk of the combined endpoint in those with *DSP*tv in the constitutive and non-constitutive NMD-competent regions.

### Event-free survival from ventricular arrhythmia based on gene region

**Figure 2:**
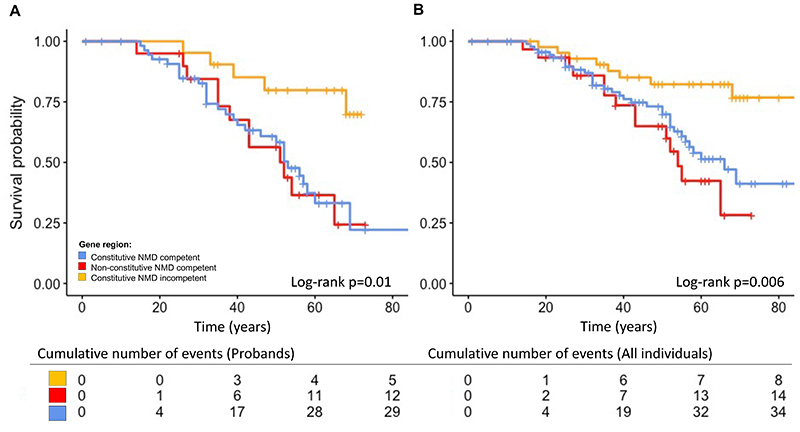
Independent life-time risk factor for ventricular arrhythmia. (A) Gene region including probands only, (B) Gene region including probands and affected family members. Time, age in years. Abbreviation: NMD, nonsense mediated decay.

Information with regard to occurrence of ventricular arrhythmia or censoring was available for 167 individuals. There were 56 probands and family members who experienced a ventricular arrhythmia during their lifetime. Univariable Cox proportional hazards models showed gene region and proband status as significantly associated with worse survival from ventricular arrhythmias (Table 3; Figure 2). Adjusting for other variables, variants in the constitutive NMD competent region (HR 2.8, 95% CI 1.3-6.0, p=0.01), non-constitutive NMD-competent region (HR 3.2, 95%CI 1.3-7.9, p=0.009) and proband status (HR 3.3, 95%CI 1.7-6.6, p=0.0006) remained significant independent life-time risk factors for ventricular arrhythmia (Table 3).

### Cutaneous phenotype

Cutaneous abnormalities were not systematically reported, however notably one family with a *DSP*tv in the non-constitutive NMD-competent region (cardiac isoform, *DSPI*) had an affected relative with hyperkeratosis and cardiomyopathy. An additional 13 individuals with *DSP*tv in the constitutive NMD-competent region and 5 in the constitutive NMD-incompetent region were reported with overt cardio-cutaneous features noted at clinical review. In eight patients (5%; 3 probands) only cutaneous abnormalities were reported, and were the sole finding in one family following an autosomal dominant inheritance pattern.

### Postmortem findings and cardiac transplant histology

Thirteen patients (8%; 10 probands) presented with SCD (Supplementary Table IV). In all 13, a postmortem investigation was performed. The mean age at death was 26 ± 11 years. Where recorded, the activity at time of death varied from exercise through to sleep. No decedent had a pre-morbid diagnosis of a cardiac condition. Nine decedents received a postmortem diagnosis of ARVC or probable ARVC. There was LV involvement in all cases and fibrosis and fatty infiltration commonly reported.

Two patients underwent a heart transplant due to end stage heart failure. Biventricular involvement was observed in both hearts, as were signs of LVNC. One heart showed ARVC with septal involvement and replacement fibrosis in both ventricles and septum. The other heart showed LVNC with notable RV involvement consisting of fatty changes and atrophy.

### Family history characteristics

Among the probands, 49 (51%) had a documented family history of cardiomyopathy, while 16 (17%) had a family history of a suspicious SCD under the age of 40 years. Of 72 family members with positive gene results included, 48 (67%) had overt disease, while 24 (33%) remained asymptomatic (mean age of 49 ± 22 years and 15 [63%] were female). There were 8 family members aged 60 years or older (60-86 years; 5 females) with no clinical evidence of disease, suggesting incomplete penetrance. By gene region, there was no statistical difference in the proportion of probands with a positive family history (constitutive NMD-competent 27 [49%], non-constitutive NMD-competent 13 [65%], constitutive NMD-incompetent 9 [43%], p=0.33).

### Literature review of previously reported *DSP*tv

Three hundred and fifteen studies were identified, 240 were screened and 185 full texts were assessed for eligibility (85 were excluded from the final qualitative synthesis, including 66 that did not report any *DSP* variant, 2 where phenotype was not provided, 2 with no full text article available, and 1 review; Supplementary Figure I). Of 98 studies (describing both disease and genotype-first cohorts) included in the final selection, a total of 105 *DSP*tv in 143 probands from apparently unrelated families were reported, including 57 nonsense, 42 frameshift, and 6 splice site variants (Supplementary Tables V-VII). All reported variants were absent or very rare (allele count ≤ 2) in gnomAD and were classified as pathogenic or likely pathogenic. One variant (p.Thr2104fs*12) was present 13 times in gnomAD however has strong evidence of pathogenicity and reported in a compound heterozygous state.

Both dominant and recessive patterns of inheritance of *DSP*tv were reported. Cascade genetic testing to confirm autosomal dominant inheritance was reported for only 19 *DSP*tv (dominant *DSP*tv) in 22 families. Families reported with autosomal dominant inheritance commonly demonstrated adult age of onset, incomplete penetrance and variable clinical expression. Of 105 reported *DSP*tv, 26 were only identified in affected individuals with homozygous or compound heterozygous inheritance. Four *DSP*tv co-occurred in trans with one of three missense *DSP* variants (p.Ala2655Asp, p.Arg2366Cys and p.Asn287Lys), each of which involved highly conserved residues within globular heads, are absent in gnomAD, and classified as likely pathogenic. There were 16 individuals with 23 *DSP*tv identified to have autosomal recessive disease, either homozygous (n=9) or compound heterozygous (n=7). In just those variants identified in a homozygous state there was only 1 (11%) in the constitutive NMD-competent region, 5 (56%) in the non-constitutive NMD-competent region and 3 (33%) in the constitutive NMD-incompetent region.

## Discussion

**Figure 3:**
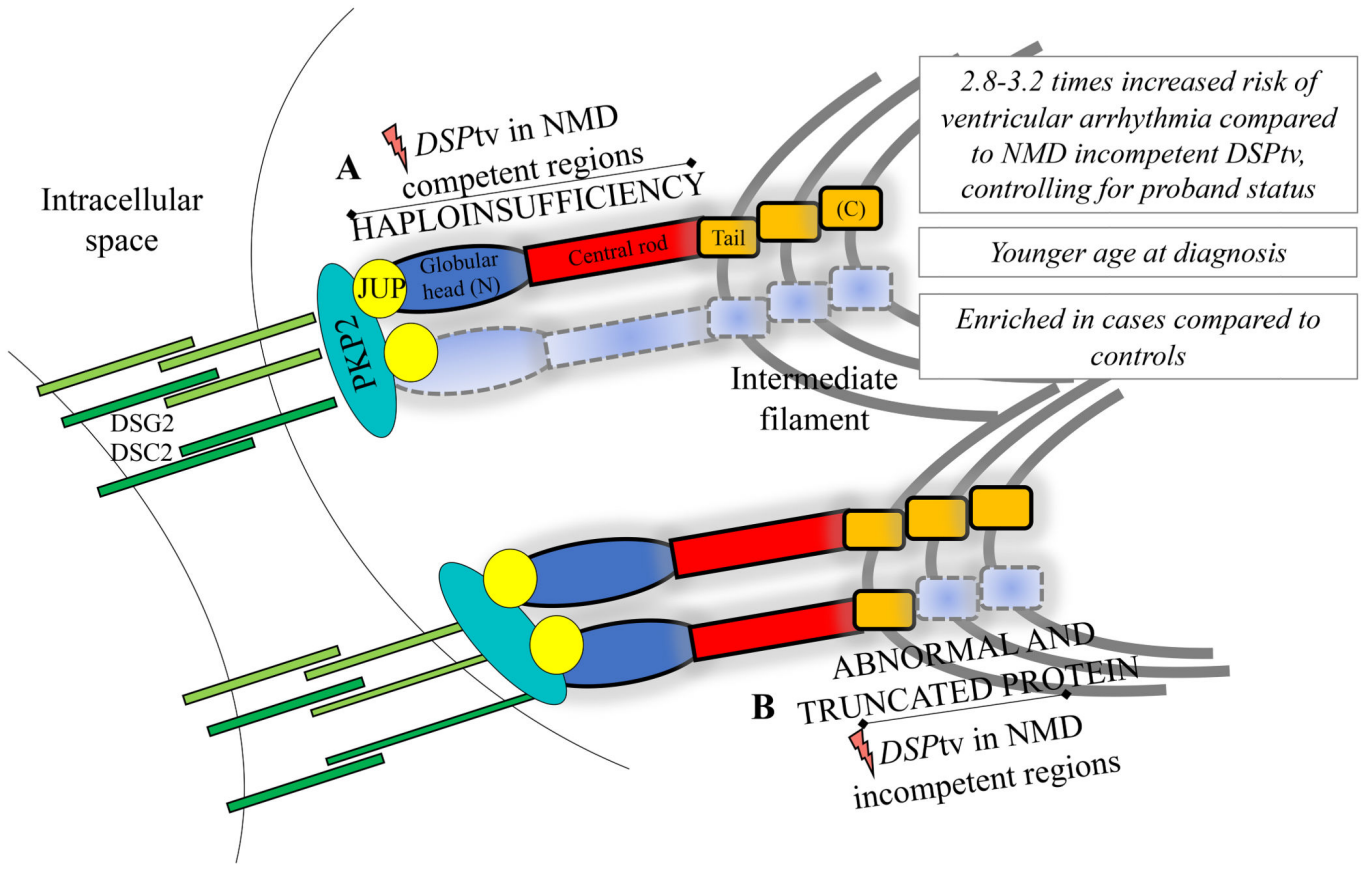
Summary of the key findings and illustration of the impact of *DSP*tv location on protein expression (for DSPI)

*DSP*tv lead to a distinct cardiomyopathy characterised by LV involvement and a high-risk of ventricular arrhythmia and SCD. We present a large international series of cases with *DSP*tv and demonstrate that the location of the *DSP*tv is a novel risk factor for ventricular arrhythmia (Figure 3). Truncating variants in the constitutive NMD-competent region were enriched in cases compared to controls, and predicted to result in NMD and haploinsufficiency of both DSPI and DSPII. Our findings highlight the importance of personalized medicine and the move towards gene-guided management of patients in the future.

Ventricular arrhythmias occur frequently in patients with *DSPtv* cardiomyopathy, with one previous study reporting 23% presenting with SCD events.^18^ In our cohort, 47% of probands had ventricular arrhythmia either at presentation or during follow-up. In addition, 14% of relatives experienced ventricular arrhythmia, including 7% as their initial presenting symptom. This included two probands who presented with resuscitated cardiac arrest without any overt structural abnormalities of the heart, supporting the notion that life-threatening electrical phenotype can precede overt cardiac structural disease.^19–21^ While we were unable to robustly ascertain clinical risk factors due to the large proportion of cases who presented with ventricular arrhythmia, i.e. without necessary pre-event clinical data, a recent series of 107 patients with any *DSP* variants (n=30 events) showed ventricular arrhythmias were associated with reduced LVEF, while premature ventricular contractions (>500 beats in 24 hours), LGE and RV dysfunction were not shown to be associated with ventricular arrhythmia.^12^ Family history of SCD has not previously been evaluated in this group, and we showed it is not associated with ventricular arrhythmia in our population.

Prior observation that *DSP*tv are predominantly associated with a left dominant form of ACM^2, 5, 22^ is in line with our findings. Recent examples of *DSP*tv presenting as recurrent myocarditis and acute myocardial infarction-like events have also been reported.^23, 24^ Females were overrepresented in our population, but otherwise shared similar clinical characteristics compared to males, except reduced indexed RV end diastolic diameter on CMR. This finding is in contrast to other reported inherited cardiomyopathy patient cohorts, where a higher prevalence of males is often reported.^25–27^ Of note, a recent report of ARVC presenting as clinical myocarditis showed disproportionately more women, with 10/11 having *DSP*tv.^28^ *DSP*tv cardiomyopathy patients frequently had low QRS voltage and negative T waves beyond V3. Low QRS voltage in limb leads have previously been shown to be associated with the presence and amount of LGE in a study of patients with ARVC.^8^ Regional wall motion abnormalities on CMR and epicardial to mid wall LGE patterns in the LV were frequently seen in our cohort. Septal LGE frequently occurs in patients with LDAC,^15^ and recent work has shown patients with *DSP* and *FLNC* ACM are more likely to have LGE, often with a ring-like pattern, compared to other DCM genotypes.^29^ Four probands were reported to have HCM, however it should be noted that all 4 probands were male, presenting in older age and 3 had mild LV hypertrophy, all characteristics previously described in the non-familial sub-group of HCM.^30^ Previous assessment of the clinical validity of *DSP* variants causing HCM failed to identify sufficient evidence of gene-disease association.^31^ While our finding remains unclear, it seems reasonable to consider these clinical diagnoses as unrelated to the *DSP*tv.

A recent systematic evaluation of cutaneous abnormalities among *DSP*tv showed all patients expressed some degree of skin or hair abnormalities, except those with *DSP*tv in the non-constitutive NMD-competent region (cardiac isoform, DSPI).^32^ Interestingly, we report one proband and their affected relative with palmoplantar keratoderma, with a *DSP*tv in the non-constitutive NMD-competent region. Another study reported 10% of *DSP*tv had cutaneous disease only, while 12% were reported to have LV dominant ACM and cutaneous disease.^5^

We show *DSP*tv localised to the constitutive NMD-competent region, corresponding to the N-terminal globular head, were enriched in patients compared to controls, and this finding was replicated in the variants identified through literature review. This region plays a critical role in organisation and assembly of the desmosomal complex by binding with plakophilin and plakoglobin. One previous report of *DSP* missense variants in patients with a clinical diagnosis of ARVC suggested a potential ‘hotspot’ N-terminal region, with 8/17 (47%) missense variants localised to the N-terminal compared to 1/28 (4%) of controls (p<0.0008).^33^ Further, they concluded *DSP*tv were significantly more prevalent in ARVC cases than controls. Indeed, a recent study also showed clustering of missense variants in the N-terminal, but reported *DSP*tv to be more evenly distributed across the gene,^12^ potentially limited by sample size. Another showed enrichment of missense variants in the spectrin repeat domain, which is part of the constitutive NMD competent region.^34^ While it seems likely that truncating variants in the NMD-incompetent region escape NMD and have a later onset and less deleterious impact, functional work to date has shown highly variable pattern of protein expression representing both haploinsufficiency and dominant negative effects.^35^ Our literature review identified biallelic *DSP*tv localized more often to the constitutive NMD-incompetent region compared to dominant *DSP*tv, suggesting that single heterozygous *DSP*tv are more likely to cause disease when occurring in the NMD-competent regions. Further, very few cases with homozygous variants in the constitutive NMD-competent region have been reported, with one example of a sib pair with severe lethal acantholytic epidermolysis bullosa, who died at 1 and 3 days respectively.^36^ It seems unlikely these infants were DSP null, given *DSP* knockout mice show embryonic lethality,^4^ suggesting expression of low-level truncated protein may be able to rescue the phenotype to some degree. Taken together, identification of a *DSP*tv in the NMD-competent regions should be considered important and may prompt gene and disease-specific adaptation and use of the ACMG/AMP criteria.^37^ We suggest *DSP*tv in this region be allocated very strong level of evidence, PVS1, when considering pathogenicity, when seen in an individual with a well characterised and concordant phenotype.

### Study Limitations

This was a large retrospective cohort study, and while it was an international effort, differences in practices and data collection by site meant some variables were incomplete. Furthermore, the event rate and data missingness precluded more detailed risk factor analyses. Diagnosis was made by the referring clinician and most recruitment was from specialised tertiary referral centres and therefore likely represents more severe phenotypes. The literature review was limited by publication bias, and inconsistent reporting of clinical, family and genetic information.

## Conclusion

We present a large international series of individuals with *DSPtv* and show gene region is a novel risk factor, specifically *DSP*tv leading to predicted NMD of truncated protein and haploinsufficiency of DSPI and/or DSPII is a risk factor for ventricular arrhythmias. By sub-typing disease by genotype there is increasing ability to offer precision medicine-based advice and therapies, and thereby improved outcomes for patients and their families.

## Supplementary Material

Supplemental Material

Supplemental Tables

## Figures and Tables

**Table 1:** Clinical characteristics by proband status and sex

		Proband status		Sex (Affected only)	
Diagnosis	Total n=178	Probands n=98	Family members n=72	Female n=86	Male n=60	p-value
Affected	146	98 (100)	48 (67)	86 (100)	60 (100)	NA
Mean age at diagnosis ± SD, years	43 ± 18	42 ± 18	45 ± 19	40 ± 17	46 ± 19	0.07
Female sex	101 (59)	57 (58)	44 (61)	NA	NA	NA
T wave inversion beyond V3	33 (24)	25 (32)	8 (13)	22 (33)	9 (18)	0.07
Low voltages	47 (35)	30 (39)	17 (29)	28 (42)	13 (27)	0.09
Premature ventricular contractions	35 (25)	23 (29)	12 (20)	25 (35)	10 (21)	0.09
Ventricular arrhythmia	56 (33)	46 (47)	10 (14)	37 (44)	19(32)	0.17
Sudden cardiac death	13 (8)	10 (10)	3 (4)	7 (8)	6 (10)	0.71
Resuscitated cardiac arrest	15 (9)	12 (12)	3 (20)	8 (10)	7 (12)	0.65
Sustained VT	19 (11)	15 (15)	4 (6)	14 (17)	5 (8)	0.24
Appropriate ICD therapy	16 (10)	16 (16)	0 (0)	13 (15)	3 (5)	0.09
End-stage heart failure	10 (8)	7 (8)	3 (5)	6 (8)	4 (8)	0.94
Heart transplant / LVAD	6 (6)	6 (6)	0 (0)	4 (5)	2 (3)	0.64
Combined endpoint	64 (38)	52 (53)	12 (17)	41 (48)	23 (39)	0.27
Myocarditis	8 (10)	6 (13)	2 (6)	2 (5)	6 (24)	0.046
**Transthoracic echocardiogram**					
LV ejection fraction	40 ± 16	35 ± 15	48 ± 13	35 ± 14	42 ± 15	0.03
LV end diastolic diameter	57 ± 9	59 ± 9	53 ± 8	58 ± 8	59 ± 10	0.56
LV end systolic diameter	44 ± 12	47 ± 12	37 ± 9	47 ± 12	44 ± 12	0.29
**CMR imaging**					
LV ejection fraction, %	42 ± 12	38 ± 12	48 ± 12	39 ± 11	43 ± 13	0.14
LV end diastolic volume, indexed	111 ± 33	117 ± 35	100 ± 23	119 ± 34	105 ± 29	0.09
LV end systolic volume, indexed	67 ± 32	73 ± 35	54 ± 20	74 ± 34	60 ± 27	0.07
RV ejection fraction, %	47 ± 12	44 ± 12	53 ± 8	47 ± 11	45 ± 12	0.46
RV end diastolic volume, indexed	89 ± 24	93 ± 25	83 ± 21	84 ± 20	100 ± 27	0.01
RV end systolic volume, indexed	48 ± 19	52 ± 19	41 ± 16	46 ± 17	56 ± 20	0.05
Regional wall motion abnormalities	44 (45)	35 (56)	9 (26)	25 (46)	19 (51)	0.64
Late gadolinium enhancement	59 (61)	40 (63)	19 (58)	31 (57)	28 (76)	0.07
LV noncompaction	22 (22)	17 (27)	5 (13)	14 (29)	8 (21)	0.42
**Gene region**					
Constitutive NMD competent	83 (57)	57 (58)	26 (54)	49 (57)	34 (57)	0.84
Non-constitutive NMD competent	27 (18)	20 (20)	7 (15)	17 (20)	10 (17)	
Constitutive NMD incompetent	36 (25)	21 (21)	15 (31)	20 (23)	16 (27)	

Data shown are n (%) or mean ± standard deviation. Abbreviations: ICD, implantable cardioverter defibrillator; LVAD, left ventricular assist device; VT, ventricular tachycardia; LV, left ventricular; CMR, cardiac magnetic resonance; RV, right ventricle Data were analysed using students t-test or chi-square test for continuous and categorial variables, respectively

**Table 2:** Cardiac investigation of affected individuals with *DSP*tv by gene region

	N	Constitutive NMD-competent	Non-constitutive NMD-competent	Constitutive NMD-incompetent	p-value
n	146	83 (57)	36 (25)	27 (18)	-
Age at diagnosis, years	143	40 ± 18	37 ± 16	52 ± 16	0.002
Female sex	146	49 (59)	17 (63)	20 (56)	0.84
Family history of disease	107	36 (57)	15 (68)	9 (43)	0.25
Ventricular arrhythmia	144	34 (41)	14 (54)	8 (22)	0.03
SCD	143	7 (9)	4 (15)	2 (6)	0.40
Resuscitated cardiac arrest	143	7 (9)	5 (23)	2 (6)	0.06
Sustained VT	143	14 (17)	1 (4)	4 (11)	0.19
Appropriate ICD therapy	143	13 (16)	3 (12)	0 (0)	0.04
Combined endpoint	144	39 (48)	15 (58)	10 (28)	0.04
Myocarditis	68	6 (18)	1 (5)	1 (7)	0.32
LV noncompaction	87	14 (26)	6 (40)	2 (11)	0.16
PVCs	119	21 (33)	4 (18)	10 (30)	0.43
**Transthoracic echocardiogram**					
LV ejection fraction	100	41 ± 16	28 ± 11	38 ± 13	0.008
LV end diastolic diameter	98	59 ± 9	57 ± 10	57 ± 7	0.71
LV end systolic diameter	77	45 ± 12	47 ± 14	46 ± 9	0.95
**CMR imaging**					
LV ejection fraction	88	41 ± 12	38 ± 13	41 ± 11	0.55
LV end diastolic volume, indexed	75	121 ± 39	114 ± 28	104 ± 22	0.13
LV end systolic volume, indexed	76	75 ± 37	69 ± 30	61 ± 24	0.27
RV ejection fraction	62	49 ± 10	45 ± 11	44 ± 14	0.35
RV end diastolic volume, indexed	56	92 ± 26	81 ± 14	93 ± 25	0.35
RV end systolic volume, indexed	55	51 ± 19	48 ± 13	51 ± 21	0.87
Late gadolinium enhancement	91	29 (64)	12 (63)	18 (67)	0.97
Regional wall motion abnormalities	91	22 (47)	6(35)	16 (59)	0.29
**Electrocardiogram**					
Sinus rhythm	81	43 (98)	16 (100)	21 (100)	0.65
PR interval	110	167 ± 43	155 ± 27	172 ± 50	0.35
QRS	108	102 ± 17	95 ± 12	97 ± 16	0.21
QTc	105	424 ± 40	417 ± 32	421 ± 39	0.76
T wave inversion beyond V3	117	21 (33)	3 (13)	7 (23)	0.14
Low voltages	114	23 (37)	8 (38)	10 (33)	0.93

Data shown are mean ± standard deviation (n = number of persons with available data) or n (%). Abbreviations: LV, left ventricle; RV, right ventricle; NMD, nonsense mediated decay; SCD, sudden cardiac death; ICD, implantable cardioverter defibrillator; PVCs, premature ventricular contractions (>500 per 24 hours). Data shown are mean ± standard deviation or n (%)

**Table 3:** Lifetime risk factors for ventricular arrhythmia for individuals with a *DSP*tv

	Univariable				Multivariable		
	N	HR	95% CI	*p* value	HR	95% CI	*p* value
Female sex	167	1.5	0.9-2.6	0.14			
Proband status	167	3.5	1.8-7.0	0.0003	3.3	1.7-6.6	0.0006
*Gene region:*	167						
Constitutive NMD competent		2.8	1.3-6.1	0.009	2.8	1.3-6.0	0.01
Non-constitutive NMD competent		3.8	1.6-9.2	0.003	3.2	1.3-7.9	0.009
Constitutive NMD incompetent		REF	-	-	REF	-	-
Family history of sudden cardiac death	110	0.7	0.3-1.7	0.44			

*DSP*tv, desmoplakin truncating variant; NMD, nonsense mediated decay; REF, reference category. For the adjusted analysis, there were total n=167 individuals included with n=56 events.
