# Supplementary material for: Variant Location Is a Novel Risk Factor for Individuals with Arrhythmogenic Cardiomyopathy Due to a Desmoplakin (DSP) Truncating Variant
Source: Circ Genom Precis Med. Author manuscript; Available in PMC 2023 Feb 24. (PMC9946166; doi:10.1161/CIRCGEN.121.003672)
Supplement: Supplemental Material [file EMS158320-supplement-Supplemental_Material.docx]

**SUPPLEMENTAL MATERIAL**

**SUPPLEMENTAL DATA**

**METHODS**

**International cohort**

An international (Australia, United Kingdom, Netherlands and United States of America) retrospective cohort of unrelated patients and family members with a *DSP*tv was assembled, comprising patients or their relatives seen in specialised cardiac genetic clinics, outpatient cardiology clinics or clinical genetics services. Cases were submitted between July 2016 and August 2018, and cross-sectional clinical data collected from the medical record at each site.

The proband was defined as the first affected in the family who underwent genetic testing with a *DSP*tv identified. Cases seen in the specialised multidisciplinary Genetic Heart Disease clinic at Royal Prince Alfred Hospital between 2002-2018 were included. International centers were included via existing collaborative networks. These included the Department of Medical Genetics, University Medical Center Groningen, Groningen, The Netherlands; Department of Medical Genetics, University Medical Center Utrecht, Utrecht, The Netherlands; Department of Medical Genetics, Maastricht University Medical Center, Maastricht, The Netherlands; Department of Medical Genetics, Amsterdam University Medical Center, Amsterdam, The Netherlands; Department of Medical Genetics, Leiden University Medical Center, Leiden, The Netherlands; Department of Medical Genetics, Erasmus Medical Center, Rotterdam, The Netherlands; Department of Medical Genetics, Radboud University Medical Center, Nijmegen, The Netherlands; Stanford Inherited Cardiovascular Diseases Group, Stanford University, California USA; and Cardiovascular Research Centre, Royal Brompton & Harefield Hospitals London UK. Members of the Australian Cardiac Genetic Testing network (ACGT) were invited to contribute probands*.* The ACGT network includes cardiac genetic professionals throughout Australia, including >90 clinicians, scientists and genetic counsellors working towards a standardised cardiac genetic testing pathway. Members were contacted from July 2016 and invited to contribute cases until August 2018. All aspects of the study were performed according to institutional human research ethics committee approval.

**Eligibility criteria**

*DSP*tv were those affecting a canonical splice-site, nonsense variants, or insertion or deletion variants leading to a frameshift. In-frame insertions and deletions were not included. All variant nomenclature adhered to the Human Genome Variation Society sequence variant nomenclature recommendations, using reference transcript NM_004415.3. Probands and family members were included irrespective of cardiac phenotype, if they carried one of the variants described above. Both deceased and living patients were included, regardless of their age. Diagnosis was recorded by the referring institution and participants were classified as probands or family members, or as clinically affected (including any cardiac phenotype such as cardiomyopathy or ventricular arrhythmias, or cutaneous phenotype) and unaffected. Genetic testing was performed by the referring institution. More detailed information is available in the supplement.

**Variant location analyses**

Genetic variant data were sought from three sources; (1) Variants identified in patients included in this study; (2) Variants submitted to ClinVar with a review status of one-star and above, that were classified as pathogenic or likely pathogenic^38^ (Data downloaded on 5th October 2018); and (3) *DSP*tv listed in gnomAD v2.1.^39^ ClinVar assertions were only those reporting cases, were evaluated to remove duplication with cohort cases and only used in the gene region analyses.

Variants were grouped in to different gene regions based on (i) whether the region is included in one or both of the 2 major isoforms *DSPI* and *DSPII* (**Figure 1**), and (ii) whether a variant in that location would be expected to trigger nonsense mediated decay (NMD). This results in three regions (**Figure 2**), a constitutive (incorporated in to both major isoforms) and NMD-competent region at the N-terminal (exons 1-22, part of exon 23, c.1-c.3582), a region that is non-constitutive (incorporated in to *DSPI* only) and NMD-competent located in the central rod domain (*DSPI/DSPIa* c.3583-c.4050 and *DSPI* c.4051-c.5379), and a region that is constitutive but NMD-incompetent at the C-terminus (exon 24; c.5324-8616).

**Genetic variant classification**

Disease variants were classified using the American College of Medical Genetics and Genomics and Association for Molecular Pathology (ACMG/AMP) standards for variant classification.^40^ In brief, the criterion pathogenic moderate (PM2) were allocated to variants with a frequency in the Genome Aggregation Database (gnomAD) of <0.04%,^40^ and pathogenic very strong (PVS1) was used for variants in the NMD competent regions. PVS1_strong was allocated to *DSP*tv in the constitutive NMD incompetent region. Use of PVS1 or PVS1_strong was used based on:

1. *DSP* is intolerant to LOF variation, with only 18% of expected LOF variants observed (LOF observed/expected score; oe 0.18), and LOF observed/expected upper bound fraction (LOEUF) of 0.26 based on gnomAD v2.1.^39^
2. *DSP*tv variants are enriched in DCM cases compared to controls, with significant enrichment in two DCM cohorts.^41^
3. *DSP*tv in the constitutive NMD incompetent region are predicted to escape NMD, however functional evidence supports truncation or alteration of this domain as having a critical impact on the protein function, hence PVS1_strong was applied.^42^ Further, re-analysis of variants reported by Mazzarotto et al.^41^ was performed based on gene region, with significant excess seen for all three regions: constitutive NMD competent (OR 37, 95%CI 16-90, p<0.0001), non-constitutive NMD competent (OR 25, 95%CI 9-73, p<0.0001) and constitutive NMD incompetent (OR 11, 95% CI 4-31, p=0.0006) compared to gnomAD v2.1 controls. There was a high etiologic fraction for all regions: 0.97, 0.96 and 0.91 respectively.

**Clinical assessment**

Clinical data from all patients (probands and family members) with a pathogenic or likely pathogenic *DSP*tv were collected retrospectively. Clinical information was obtained by review of the medical record and cardiac investigations from the referring institution. Review of the genetic result, ECG, transthoracic echocardiogram, 24-hour ambulatory ECG (Holter) monitoring, cardiac magnetic resonance (CMR) imaging, three-generation pedigree, postmortem report and correspondence from the treating geneticist and/or cardiologist were reviewed by the study team where possible.

**Diagnosis and clinical definitions**

The LV was considered to be involved when one or more of the following was present: LV ejection fraction <55%, presence of LV late gadolinium enhancement (LGE) or intramyocardial fat (including septum) on CMR imaging or pathologic abnormalities found on autopsy after sudden cardiac death (SCD). Cutaneous abnormalities were defined as palmoplantar keratoderma and/or woolly hair. Cutaneous abnormalities were not systematically investigated, but included if noted in the medical record. Regional wall motion abnormalities included those reported in either the right or left ventricle. Premature ventricular contractions were defined as >500/24 hours. Those with a primary ventricular arrhythmia phenotype had a high burden of premature ventricular complexes on Holter monitoring (>10% over 24 hours) or resuscitated cardiac arrest in the absence of structural and functional abnormalities of the myocardium assessed at cardiac evaluation. A composite outcome of ventricular arrhythmia included SCD, resuscitated cardiac arrest, appropriate implantable cardioverter-defibrillator (ICD) therapy, or sustained ventricular tachycardia. Appropriate ICD therapy was defined as anti-tachycardia pacing, or an ICD discharge for termination of ventricular tachycardia or fibrillation. SCD in probands was defined as sudden death in an otherwise healthy individual of any age within 1 hour after the onset of symptoms, or when unwitnessed, within 24 hours after the individual was last seen in good health. Family history of SCD included those with a suspicious death of a first-degree relative aged less than 40 years.

**Literature review**

We conducted a literature review of all *DSP*tv reported in PubMed (accessed on June 3, 2019). We selected *DSP*tv reported in publications through a search using the terms “*DSP*” or “desmoplakin” in combination with “mutation” or “variant” in title and/or abstract. No limitations were placed on language, type, or date of publications. This selection was matched with variants reported in HGMD Pro and the ARVC database.^43^ We selected all variants in patients with available clinical information. Particular care was taken in reviewing the cardiologic and ectodermal manifestations, age of patients at evaluation and diagnosis, additional performed genetic tests, family history and cascade screening, immunohistochemical, ultrastructural or functional analysis.

**Statistical analysis**

Data were analysed using RStudio (version 1.2). One-way analysis of variance or *t*-test was used for comparing continuous variables and chi-square or Fisher exact test for categorical variables. Multivariable Cox proportional hazards models were used to assess freedom from ventricular arrhythmia, using time since birth as the time variable. For patients with ventricular arrhythmia, time to first event was used. Where there was no ventricular arrhythmia, the date of the last known cardiac evaluation was used for censoring. Hazard ratios and 95% confidence interval (CI) were calculated. To account for family-clustering in the data, analyses were also repeated in a proband only dataset (data not shown). Variables reaching p<0.05 in univariate Cox regression were included in the multivariate Cox regression model, using a backward step-wise approach.

**RESULTS**

**Literature review of reported *DSP*tv**

Isolated cardiomyopathy, cardiocutaneous disease, and isolated cutaneous disorders were associated with both mono- and bi-allelic *DSP*tv. Cardiac manifestations reported included DCM, ACM (either right, left or biventricular involvement), SCD, early onset cardiac failure, and peripartum cardiomyopathy. Cutaneous abnormalities were congenital or appeared later, most commonly in the first year of life, and included palmoplantar keratoderma, woolly hair, hypotrichosis, alopecia, abnormally shaped teeth, enamel defects in both the deciduous and permanent dentition, tong erosion, nail dystrophy, and lethal acantholytic epidermolysis bullosa, with more severe manifestations seen in bi-allelic disease. Affected patients harboring homozygous or compound heterozygous *DSP*tv always presented with skin abnormalities, more often in association with an early onset cardiomyopathy (15 [75%] families) than as an isolated trait (5 [25%] families). As many patients without reported cardiomyopathy were evaluated in their first decade, a later-onset cardiomyopathy could not be ruled out in these families. When families were ascertained via a proband <10 years of age, early onset cardiomyopathy associated with cutaneous disease was the most prevalent phenotype, and disease was caused by biallelic *DSP*tv.

**Supplemental Tables** (see separate Excel file)

**Supplemental Table I:** *DSPtv* identified in the patient cohort (see separate Excel file)

**Supplemental Table II:** ClinVar variants included as cases in the case-control analysis

**Supplemental Table III:** gnomAD variants used as controls in the case-control analysis

**Supplemental Table IV:** Sudden cardiac death cohort postmortem findings

**Supplemental Table V:** Literature review frameshift variants

**Supplemental Table VI:** Literature review splice site variant

**Supplemental Table VI:** Literature review splice site variant

**Supplemental Table VII:** Literature review nonsense variants

**Supplementary Figure I:** Overview of the systematic review search criteria

^^
